# Willingness to Vaccinate Against Herpes Zoster and Its Associated Factors Across WHO Regions: Global Systematic Review and Meta-Analysis

**DOI:** 10.2196/43893

**Published:** 2023-03-09

**Authors:** Qiang Wang, Liuqing Yang, Lan Li, Chang Liu, Hui Jin, Leesa Lin

**Affiliations:** 1 Department of Epidemiology and Health Statistics School of Public Health Southeast University Nanjing China; 2 Department of Infectious Disease Epidemiology London School of Hygiene and Tropical Medicine London United Kingdom; 3 Centre for Digital Public Health in Emergencies Institute for Risk and Disaster Reduction University College London London United Kingdom; 4 Key Laboratory of Environmental Medicine Engineering Ministry of Education School of Public Health Southeast University China; 5 Laboratory of Data Discovery for Health Hong Kong Science Park Hong Kong China; 6 WHO Collaborating Centre for Infectious Disease Epidemiology and Control, School of Public Health, Li Ka Shing Faculty of Medicine The University of Hong Kong Hong Kong China

**Keywords:** herpes zoster vaccine, willingness, associated factors, systematic review

## Abstract

**Background:**

A life-course immunization approach would enhance the quality of life across all age groups and improve societal well-being. The herpes zoster (HZ) vaccine is highly recommended for older adults to prevent HZ infection and related complications. The proportions of willingness to receive the HZ vaccine varies across countries, and various kinds of factors, including sociodemographics and individual perceptions, influence the willingness to vaccinate.

**Objective:**

We aim to estimate the HZ vaccination willingness rate and identify factors associated with vaccine uptake willingness across all World Health Organization (WHO) regions.

**Methods:**

A global systematic search was performed on PubMed, Web of Science, and the Cochrane Library for all papers related to the HZ vaccine published until June 20, 2022. Study characteristics were extracted for each included study. Using double arcsine transformation, vaccination willingness rates with 95% CIs were pooled and reported. The willingness rate and associated factors were analyzed by geographical context. Associated factors were also summarized based on Health Belief Model (HBM) constructs.

**Results:**

Of the 26,942 identified records, 13 (0.05%) papers were included, covering 14,066 individuals from 8 countries in 4 WHO regions (Eastern Mediterranean Region, European Region, Region of the Americas, and Western Pacific Region). The pooled vaccination willingness rate was 55.74% (95% CI 40.85%-70.13%). Of adults aged ≥50 years, 56.06% were willing to receive the HZ vaccine. After receiving health care workers’ (HCWs) recommendations, 75.19% of individuals were willing to get the HZ vaccine; without HCWs’ recommendations, the willingness rate was only 49.39%. The willingness rate was more than 70% in the Eastern Mediterranean Region and approximately 55% in the Western Pacific Region. The willingness rate was the highest in the United Arab Emirates and the lowest in China and the United Kingdom. The perception of HZ severity and susceptibility was positively associated with vaccination willingness. The perceived barriers to vaccination willingness (main reasons for unwillingness) included low trust in the effectiveness of the HZ vaccine, concerns about safety, financial concerns, and being unaware of the HZ vaccine’s availability. Older individuals, those having lower education, or those having lower income levels were less likely to willing to be vaccinated.

**Conclusions:**

Only 1 in 2 individuals showed a willingness to be vaccinated against HZ. The willingness rate was the highest in the Eastern Mediterranean Region. Our findings show the critical role HCWs play in promoting HZ vaccination. Monitoring HZ vaccination willingness is necessary to inform public health decision-making. These findings provide critical insights for designing future life-course immunization programs.

## Introduction

A life-course immunization approach would enhance the quality of life across all age groups and improve societal well-being through a healthier population [1,2]. Older adults are at high risk of being ill, being hospitalized, and dying from many vaccine-preventable diseases [3]. The World Health Organization (WHO) and health authorities in various countries have recommended several vaccines for older adults, including the seasonal influenza vaccine; pneumococcal vaccine; hepatitis B vaccine; the tetanus, diphtheria, and pertussis (Tdap) vaccine booster; and, most recently, COVID-19 vaccines [2]. The herpes zoster (HZ) vaccine is highly recommended for older adults for reducing the incidence of HZ [4].

Caused by varicella zoster virus (VZV), HZ is a distinctive syndrome occurring when immunity to VZV declines due to age or immunosuppression [5]. It presents a disproportionate risk to adults aged 50 years and over, with estimated incidence rates reaching 5.2-10.9 cases per 1000 person-years [6]. Due to the potential for serious complications, postherpetic neuralgia (PHN), neurological sequelae, HZ ophthalmicus with eye involvement, and disseminated disease, this disease has a significant impact on the quality of life [6,7]. It was reported that 5%-30% of patients with HZ are likely to develop PHN with persistent pain for more than 1 month [7].

Existing evidence supports the substantial efficacy of the HZ vaccine; a meta-analysis showed that the efficacy of the adjuvant recombinant subunit HZ vaccine is 94% when compared with the placebo [8]. Additionally, HZ vaccination was found to be cost-effective versus no vaccination for older adults in high-income countries based on health economics evaluations [9,10].

Currently, 2 types of HZ vaccines (Zostavax and Shingrix) are commonly used in over 60 countries, being recommended to adults aged ≥50 years or adults aged ≥18 years with an immunocompromised state [11]. Noticeably, HZ vaccination uptake is relatively low worldwide. In the United States, 24.1% of adults aged ≥50 years received an HZ vaccine in 2018 [12]. The coverage rates among older adults in Australia and Canada were reported to be lower than 10% [13,14]; only 1.8% of adults over 65 years old received the HZ vaccine in Turkey [15].

Vaccine uptake is positively associated with the willingness to vaccinate. The willingness to receive the HZ vaccine has been surveyed in previous studies [16-18]. However, the proportions of the willingness to receive the HZ vaccine varies across countries, ranging from 17% (China) to 90% (Australia) [16,17]. Importantly, vaccination willingness is influenced by multiple factors, which include sociodemographic, cognitive, psychologic, and politics-and-culture contexts [19]. According to recent evidence, various kinds of factors, including sociodemographics (age and income level) and individual perceptions (perception of disease susceptibility), influence HZ vaccination willingness [16-18]. Additionally, these associated factors may differ across different regions.

Summarizing and understanding the willingness to receive the HZ vaccine and its associated factors is thus essential for obtaining insights into intervention strategies to improve its uptake among older adults. It is also necessary to explore methods mitigating vaccine hesitancy among older adults, which is also an indispensable part of life-course immunization practices. However, there has only been scant attention paid to HZ vaccination willingness in the research literature. To fill this gap, this systematic review seeks to summarize the up-to-date evidence on the HZ vaccination willingness rate and identify its associated factors.

## Methods

### Study Design

The Preferred Reporting Items for Systematic Reviews and Meta Analyses (PRISMA) statement was used for this systematic review [20].

### Search Strategy and Selection Criteria

A systematic search was conducted in 3 electronic databases (PubMed, Web of Science, and the Cochrane Library) using the following search terms: [“Herpes Zoster” OR “Herpesvirus 3” OR shingles OR zoster OR “varicellovir*” OR hhv3 OR hhv-3 OR varicella-zoster OR postherpetic] AND [“vaccin*” OR “immuniz*” OR “inocul*”]. All studies published from the inception of the databases until June 20, 2022, were included. After combining all search records, duplicate records were identified and removed. Titles and abstracts were then screened for relevance, and full texts of the included records were retrieved and reviewed.

The screening process was independently performed by 2 reviewers (authors QW and LQY) with the following inclusion criteria: (1) cross-sectional surveys or cohort studies and (2) relevance to HZ vaccine attitudes, intentions, willingness, and acceptance. There were no restrictions on language or paper type. Papers were excluded if they met any of the following criteria: (1) willingness data were not reported, (2) insufficient data were provided for pooling (at least 2 of the 3 following topics were absent: total number of surveyed individuals, number of individuals willing to receive the HZ vaccine, and willingness rate), (3) they were duplicate data or surveys, or (4) they were experimental and observational studies that reported the willingness rate after interventions. Any disagreement was resolved by discussions with a third reviewer (author LL). The review protocol is available in the International Prospective Register of Systematic Reviews (PROSPERO id: CRD42022348426).

### Data Abstraction and Quality Assessment

For each included study, we extracted the following data: title, first author, journal name, published date, paper type, sampling method, study setting, study period, study population, study location, and inquiry questions (were there any assumptions, for example, free vaccination was provided). The number of surveyed individuals, the number of individuals who accepted HZ vaccination, and the willingness rate were also extracted for pooling. Additionally, influencing factors examined in each study were abstracted.

The Strengthening the Reporting of Observational Studies in Epidemiology (STROBE) statement was used to assess the quality of the included papers [21]. Data abstraction and quality assessment were conducted by QW and LQY independently. A third researcher, LL, resolved disputes when the data extracted or quality assessment scores for papers were not consistent between the 2 initial reviewers.

### Statistical Analysis

Using double arcsine transformation on 2 variables, number of surveyed individuals and number of individuals accepting HZ vaccination, we calculated and reported the pooled willingness rate and the 95% CI [22,23]. If these data were not included in the study, the number of individuals accepting HZ vaccination could be calculated by multiplying the total number by the willingness rate. Due to high heterogeneity, DerSimonian-Laird random effects were used in the calculation.

Stratified subgroup analysis was subsequently performed to explore the causes of heterogeneity according to the study characteristics (eg, sampling method, study setting, study period, and study population). Studies that shared the same characteristics were grouped together (eg, surveyed using the random sampling method or the convenience sampling method); when the characteristic was unclear, the study was labeled as “not mentioned” in the subgroup analysis. For each subgroup, the willingness rate was pooled. To examine the potential impact of the COVID-19 pandemic on the willingness to receive the HZ vaccine, we divided the study period into 2 subperiods, before the pandemic and during the pandemic. The study locations were categorized by their WHO regions and economic levels [24,25]. Due to the limited availability of data, it was difficult to further estimate the willingness rate by age group. We only reported the willingness rate among individuals aged ≥50 years in further analysis of age.

The willingness rate and its associated factors were examined by geographical context in order to explore potential differences. We also classified factors based on Health Belief Model (HBM) constructs. This method was previously used to identify factors associated with seasonal influenza vaccination, human papillomavirus vaccination, and COVID-19 vaccination [26-28]. Following the HBM construct, the influencing factors were summarized and classified according to the perceived susceptibility and severity of HZ, the perceived benefits of and barriers to vaccination willingness (reasons for vaccination willingness or unwillingness), modified factors, and cues to action. We evaluated the publication bias using the funnel plot [29]. The publication bias was not adjusted because of the presence of substantial heterogeneity [30]. All analyses were conducted in STATA version 14.0 (StataCorp) and Microsoft Excel 2016.

## Results

### Search Results and Quality Assessment

**Figure 1:**
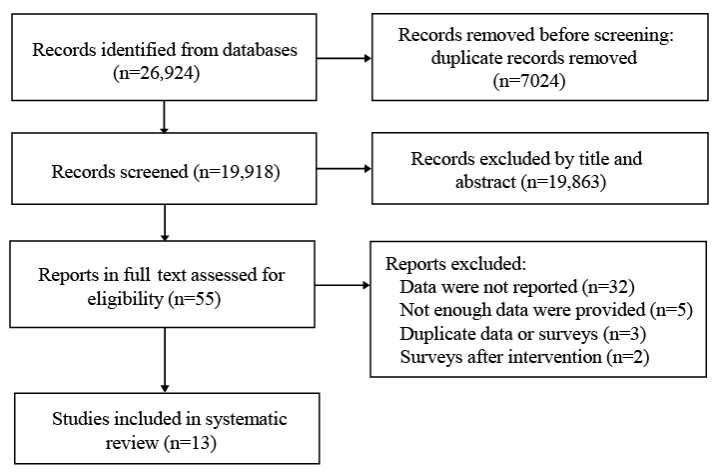
Flowchart of paper selection.

The initial search identified 26,942 papers. After deduplication and screening by title and abstract, 55 (0.20%) papers were screened by full text. A total of 13 (23.6%) papers were included in this review. The detailed selection process is illustrated in Figure 1. Of the 13 studies, 10 (76.9%) were empirical studies [16,18,31-38], 2 (15.4%) were meeting abstracts [17,39], and 1 (7.7%) was a letter [40]. All 13 studies belong to cross-section surveys. The final analysis included 14,066 individuals from 8 countries (Australia, Canada, China, France, South Korea, the United Arab Emirates, the United Kingdom, and the United States). The 13 studies were conducted in 4 WHO regions: the Eastern Mediterranean Region, the European Region, the Region of the Americas, and the Western Pacific Region. Study populations were adults aged ≥50 years in 10 (76.9%) studies, adults aged ≥18 years in 2 (15.4%) studies, and not specified in the remaining 1 (7.7%) study. The quality assessment scores of the included studies ranged from 10 to 20, with an average of 16.30 (SD 3.86). Detailed study characteristics and quality assessment results of the included studies are provided in Multimedia Appendix 1, Tables S1 and S2.

### Vaccination Willingness Rate

The pooled HZ vaccination willingness rate was estimated to be 55.74% (95% CI 40.85%-70.13%). The asymmetry plots in the funnel plot indicated that there might be publication bias (Multimedia Appendix 1, Figure S1). It was estimated that 56.06% (95% CI 37.26%-74.02%) of adults aged ≥50 years were willing to receive an HZ vaccine. There were no significant differences between pooled willingness rates from surveys in the hospital context (58.29%, 95% CI 34.35%-80.33%) and surveys in the community context (53.45%, 95% CI 33.07%-73.26%), as seen in Multimedia Appendix 1, Figure S2. The willingness rate was 71.90% (95% CI 67.42%-75.99%) in the Eastern Mediterranean Region and 55.68% (95% CI 22.03%-86.67%) in the Western Pacific Region. The willingness rate was estimated to be 60.18% (95% CI 47.53%-72.18%) in high-income economies and 23.97% (95% CI 22.22%-25.77%) in upper-middle-income economies. Under health care workers’ (HCWs) recommendations, 75.19% (95% CI 54.61%-91.07%) of individuals were willing to receive the HZ vaccine, which was higher than the willingness rate among individuals (49.39%, 95% CI 30.42%-68.45%) without HCWs’ recommendations.

### Rate and Associated Factors by Geographical Context

#### Eastern Mediterranean Region

Only 1 (7.7%) study was conducted in the United Arab Emirates [35], which reported a willingness rate of 71.9%, as shown in Multimedia Appendix 1, Figure S3. This study found that persons who were unsure about their chickenpox infection history were less likely to receive the HZ vaccine. In this study, the top 3 reasons respondents provided explaining their unwillingness were “prefers to take medication when sick,” “side effects of vaccine,” and “not at risk since I am healthy.”

#### European Region

Of the 13 studies, 2 (15.4%) reported findings from surveys conducted in France (68.91%) [31] and the United Kingdom (34.62%) [36]. In France, women and persons in a relationship/married were more likely to vaccinate themselves against HZ. This study also identified that persons who agreed with the statements “HZ causes pain,” “HZ is always severe,” or “vaccination is a good prevention tool against HZ” preferred to receive the HZ vaccine. In the United Kingdom, Nicholls et al [36] reported the association between vaccine hesitancy using the Vaccination Attitudes Examination (VAX) scale and the 5C scale. Lower scores on “collective responsibility” on the 5C scale and higher scores on “concerns” on the VAX scale were associated with a lower likelihood of receiving the HZ vaccine.

#### Region of the Americas

A total of 4 (30.8%) studies were conducted in the Region of the Americas, including the United States (n=3, 75%) [33,34,40] and Canada (n=1, 25%) [32]. The willingness rates in the United States and Canada were 53.28% and 55.01%, respectively. People in the United States with a higher income were more likely to receive an HZ vaccine [34]. Two surveys in the United States reported reasons for the refusal of the HZ vaccine; the top 3 reasons in Lu et al’s [34] study were “the vaccination was not needed,” “not a risk, healthy, or high immune,” and “not trusting doctors or medicine”; according to Funovits et al [40], the top 3 reasons were “believes vaccine is not important,” “not covered by insurance,” and “would like to obtain primary care physician recommendations.”

#### Western Pacific Region

There were 6 (34.6%) surveys carried out in Australia (n=1, 16.7%), South Korea (n=1, 16.7%), and China (n=4, 66.6%). More than 85% of individuals were willing to accept the HZ vaccine in Australia and South Korea, considerably higher than that observed in China (35.13%). As shown in surveys in South Korea [37], younger people were more likely to accept the HZ vaccine. Individuals with a college education were more likely to accept the HZ vaccine than those with incomplete high school education. In this study, the top 3 reasons for unwillingness were “low perceived risk of developing HZ,” “concerns about the adverse effects following immunization,” and “concerns about the vaccination cost” [37].

Of the 4 studies in China, 2 (50%) were conducted in Hong Kong and 2 (50%) in Shanghai City. In the studies in Hong Kong, the most frequent reason listed for refusal was “they were unaware of its availability” [18]. According to Lu et al [16], in Shanghai City, younger people, people with higher monthly incomes, people who know the elderly are susceptible to HZ, and people with lower vaccine hesitancy levels were more willing to receive the vaccine. Qiu et al [38] reported that people who know about the HZ vaccine, are concerned with getting an HZ infection themselves, and who believe the vaccine can prevent HZ have a greater likelihood of receiving the HZ vaccine [38].

### Associated Factors Using HBM Constructs

Information about how factors associated with vaccination willingness relate to HBM constructs is provided in Table 1 and Multimedia Appendix 1, Table S3.

**Table 1:** Description of influencing factors using HBM^a^ constructs.

HBM framework			Factors		References
**Individual perceptions**					
	Perceived susceptibility and severity of HZ^b^	Perception of HZ infection susceptibility and severity		[16,31,38]	
	Perceived barriers to vaccination willingness	Low trust to the effectiveness of HZ vaccines, unawareness of HZ vaccine availability, concerns about side effects and safety, and cost		[18,34,35,37,39]	
**Modifying factors**					
	Sociodemographics	Age Education level Gender Income level Marital status Insurance Race Nationality Occupation		[16,31,33,34,37,38,40] [16,31,34,37,38,40] [31,34,37,38,40] [16,34,37,38] [31,34,36] [16,34,35] [34] [35] [35]	
	Knowledge, attitude, beliefs, and prior experience	Confidence in the effectiveness of the HZ vaccine Knowing the HZ vaccine Receiving influenza vaccination HZ infection history With chronic diseases		[31,38] [31,38] [34,37] [31,35,37,38] [35,38]	
**Cues to action**					
	Interpersonal relationships	Knowing someone with a history of HZ		[31,37,38]	
	Community	Doctors’ recommendations		[17]	

^a^HBM: Health Belief Model.

^b^HZ: herpes zoster.

#### Perceived Susceptibility and Severity of Herpes Zoster

Of the 13 studies, 2 (15.4%) reported that the perception of disease severity is positively associated with vaccination willingness [31,38], while 1 (7.7%) study found a positive relationship between perceived infection susceptibility and vaccination willingness [16].

#### Perceived Benefits of and Barriers to Vaccination Willingness

Of the 13 studies, 5 (38.5%) explored reasons for vaccination unwillingness (perceived barriers to willingness). Low trust in the effectiveness of the HZ vaccine, concerns about safety, low perception of disease risk, financial concerns, and unawareness of the HZ vaccine’s availability contributed to the unwillingness to receive the HZ vaccine [18,34,35,37,40].

#### Modified Factors

Modified factors included (1) sociodemographics and (2) knowledge, attitude, beliefs, and prior experience in the analysis.

### Sociodemographics

Age (7/13, 53.8%), education level (6/13, 46.2%), gender (5/13, 38.5%), and income level (4/13, 30.8%) were the most frequent factors reported in the included studies. Of the 13 studies, 3 (23.1%) found a nonsignificant association between age and vaccination willingness [31,34,40], whereas 4 (30.8%) reported a significant relationship [16,33,37,38]. Nonsignificant relationships between education level and willingness were reported in 3 (23.1%) studies [31,34,40], while the other 3 (23.1%) studies found that a higher education level is associated with higher willingness rates [16,37,38]. In addition, 4 (30.8%) studies showed a nonsignificant association between gender and vaccination willingness [34,37,38,40]; however, 1 (7.7%) study reported that males are more likely to receive the HZ vaccine [31]. It was reported that individuals with higher income levels are more likely to receive the HZ vaccine [16,34], but 2 (15.4%) studies found no significant association between income level and willingness rate [37,38]. Additionally, 1 (7.7%) study reported a significant association between marital status and vaccination willingness [31], whereas 2 (15.4%) studies found no significant association between these factors [34,36].

### Knowledge, Attitude, Beliefs, and Prior Experience

We found that confidence in the effectiveness of the HZ vaccine is positively associated with the willingness to receive the HZ vaccine [31,38]. Individuals who knew about the HZ vaccine were more likely to receive it [38], a finding that was not observed in another study [31]. Having a history of HZ infection was positively correlated with vaccination willingness [38]; however, 2 (15.4%) studies reported only nonsignificant associations between infection history and vaccination willingness [31,37]. Influenza vaccination history was not found to be an influencing factor for HZ vaccination acceptance [34,37]. In addition, 1 (7.7%) study reported that individuals without chronic diseases are more likely to receive the HZ vaccine [38], whereas another study did not find a significant association between these factors [35].

### Cues to Action

Doctors’ recommendations to receive the HZ vaccine were positively associated with vaccination willingness [17]. The experience of knowing someone with a history of HZ was positively associated with vaccination willingness [38], albeit this relationship was found to be insignificant in 2 (15.4%) studies [31,37].

## Discussion

### Principal Findings

This systematic review demonstrated that the pooled HZ vaccination willingness rate is 55.74% worldwide. The main reasons for the unwillingness to receive the HZ vaccine include low trust in the effectiveness of the HZ vaccine, concerns about safety, low perceptions of disease risk, financial concerns, and unawareness of the availability of the HZ vaccine. Our findings showed that HCWs’ recommendations are correlated with a greater likelihood of receiving an HZ vaccine.

A large variability in HZ vaccination rates was found; however, certain patterns could still be deduced. Higher vaccination acceptance rates were observed in countries with an earlier license date of the HZ vaccine. From 2006 onward, countries with developed economies gradually licensed the HZ vaccine, such as the United States, France, Australia, South Korea, and the United Kingdom [11]. People living in these countries may have a greater awareness of HZ vaccine availability and may have received more frequent recommendations from HCWs to receive the vaccine. The willingness rate in China was considerably lower than that in these countries. HZ vaccines were first licensed in China in 2019 [11]. Less than one-third of the participants knew about the availability of the HZ vaccine in Shanghai, China [38]. The lack of awareness of HZ vaccine availability among the public may contribute to the low vaccination willingness rate in China. However, although the United Kingdom approved the HZ vaccine in 2006, the observed vaccination willingness rate remains low. The willingness rate might also be associated with the incidence of HZ across the countries. According to van Oorschot et al [6], the cumulative incidence of HZ in South Korea and Australia (more than 10 cases per 1000 population) is considerably higher than that in Canada, China, and France (between 2.9 and 8.67 cases per 1000 population) [6]. Thus, the higher incidence rate might result in individuals having an increased perception of susceptibility to HZ infection.

Recommendations from HCWs were associated with a higher HZ vaccination willingness rate; the impact of HCWs’ recommendations on vaccine uptake has been observed in previous studies [41,42]. In turn, this channel can be an effective communication strategy to tackle vaccine hesitancy [43]. Glenton et al [44] summarized HCWs’ views and experiences of communicating about vaccination with individuals over 50 years old and found that misinformation, fears, and concerns about vaccines are commonly voiced among older adults during their communications with HCWs. Hence, HCWs’ attitudes and practices regarding the HZ vaccine are crucial. Reassuringly, HZ vaccination was reportedly recommended by 65.6% of HCWs in Italy [45]. A study in the United States reported that more than 90% of HCWs reported were willing to recommended HZ vaccination to their patients [46]. However, there remains a gap in relevant data in countries with upper-middle-income economies; therefore, more studies in upper-middle-income economies should be carried out to determine provider willingness to recommend an HZ vaccine.

Sociodemographic factors, while inconclusive, are essential to understanding and predicting HZ vaccination willingness rates. Individuals with lower education and income levels might be less likely to receive the HZ vaccine. Similar results have been observed in previous reviews of factors associated with influenza vaccination uptake and COVID-19 vaccination willingness among older adults [47,48]. Low socioeconomic status can be an important barrier to vaccination for older adults. Worryingly, the price of the HZ vaccine is considerably higher (about 10 times) than that of other vaccines, such as the influenza vaccine and the Tdap vaccine [49,50]. Along with a low perception of disease risk, low trust in the HZ vaccine, and awareness of HZ vaccine availability, the price of the vaccine might also represent a significant barrier to vaccination willingness.

### Implications for Policy and Practice

Our findings have important implications for developing intervention strategies for increasing HZ vaccine acceptance across multiple levels. Countries with earlier licensing dates should pay attention to methods to improve individuals’ confidence in the HZ vaccine and decrease their complacency. It is essential for countries with later licensing dates, such as China, to increase the awareness of the availability of the HZ vaccine first. Embracing the idea of communication strategies could be a potentially effective way to improve the acceptance of the HZ vaccine. One such strategy is engaging HCWs, as HCWs play a crucial role in older peoples’ vaccination decisions [44]. Active recommendations from HCWs could be an effective way to increase vaccine awareness and willingness, as well as vaccine confidence. Using social media, such as posting content on Facebook or YouTube, is another feasible way to educate people about HZ vaccination [51]. At the individual level, our findings could facilitate greater awareness of people with identified factors for unwillingness (eg, age and income) and may help create tailored interventions for these populations using behavioral change strategies and techniques [52]. Our findings also indicate that some challenges exist toward the practice of life-course immunization, such as individuals being might face some challenges. Due to a lack of information, individuals might need to be aware of age-specific immunizations, which further emphasizes that the role of HCWs in promoting life-course immunization is vital and critical.

### Limitations

Several limitations of this review were identified. First, the representativeness of the samples in the included studies was unclear. Only 3 of the 13 studies used a random sampling method. However, stratified subgroup analysis was adopted to mitigate this potential bias. Although no significant differences were observed among sampling methods, the results need to be interpreted with caution. Second, a lack of studies in this area limited the diversity and representativeness of our review findings. Since most studies were performed in countries with high-income economies, more studies need to be carried out in less economically developed countries. Additionally, some associated factors might not be reported, because the included studies did not explore them. For example, vaccination policy and smoking habits were determined to be associated with influenza vaccination willingness [53,54]; trust in the government and information exposure were associated with the COVID-19 vaccine [28,55]. These could be explored in future research.

### Conclusion

Only 1 in 2 individuals showed a willingness to be vaccinated against HZ. The willingness rate was the highest in the Eastern Mediterranean Region. A higher acceptance rate could be generally observed in countries licensing HZ vaccines earlier or having higher HZ incidence, such as Australia and South Korea. The reasons for unwillingness included low trust in the effectiveness of the HZ vaccine, concerns about safety, low perceptions of disease risk, financial concerns, and unawareness of the availability of the HZ vaccine. Our findings showed the critical role HCWs play in promoting HZ vaccination. Countries with earlier vaccine licensing dates and high HZ incidence should improve individuals’ confidence in the vaccine and decrease their complacency. It is necessary for countries with later licensing dates to prioritize the improvement of the awareness of HZ vaccine availability. Monitoring HZ vaccination willingness is necessary to inform public health decision-making. These findings will also be of interest to the practice of life-course immunization.

## Appendix

Multimedia Appendix 1 Supplementary material.

## Abbreviations

HBM: Health Belief Model
HCW: health care worker
HZ: herpes zoster
PHN: postherpetic neuralgia
Tdap: tetanus, diphtheria, and pertussis
VAX: Vaccination attitudes examination
VZV: varicella zoster virus
WHO: World Health Organization

## Data Availability

The data underlying this paper are available within the paper and in Multimedia Appendix 1.
