# Supplementary material for: Willingness to Vaccinate Against Herpes Zoster and Its Associated Factors Across WHO Regions: Global Systematic Review and Meta-Analysis
Source: JMIR Public Health Surveill. 2023 Mar 9;9:e43893. doi: 10.2196/43893 (PMC10037179; doi:10.2196/43893)
Supplement: Multimedia Appendix 1 [file publichealth_v9i1e43893_app1.docx]

**Willingness to vaccinate against herpes zoster and its associated factors across WHO regions: a global systematic review and meta-analysis**

Table S1. Characteristics of 13 included studies

Table S2. Quality assessment of 13 included articles

Table S3. Summarized influencing factors explored in included articles

Figure S1. Funnel plot

Figure S2. Vaccination willingness rates among different subgroups ^a^

Figure S3. Estimated HZ vaccination willingness rate worldwide ^a^

Reference

**Table 1.** **Characteristics of 13 included studies**

| **Ref** | **First Author** | **Journal** | **Article type** | **Study setting** | **Sampling method** | **N** | **Study population** | **Study period** | **Country** | **Under doctors' recommendation** |
| --- | --- | --- | --- | --- | --- | --- | --- | --- | --- | --- |
| 1 | Corinne Del Signore | Vaccine | Article | Hospital | NA | 907 | Patients aged 65 and over | January, 2018 to March, 2019 | France | No |
| 2 | J.C.B. Litt | International Journal of Infectious Diseases | Meeting abstract | Hospital | Random sampling | 1,330 | Patients aged between  60 and 85 | NA | Australia | Yes |
| 3 | D M MacDougall | BMJ open | Article | Community | Random sampling | 4,023 | Adults | NA | Canada | Yes |
| 4 | Nour A. Baalbaki | Infectious Diseases and Therapy | Article | Community | Convenience sampling | 373 | Adults aged ≥ 50 years | June to August, 2018 | The  United States | No |
| 5 | Anthony CY Lam | Hong Kong Medical Journal | Article | Hospital | Convenience sampling | 408 | Patients aged 50  years or above | 24 July to 12 August, 2015 | Hong Kong, China | No |
| 6 | Peng-jun Lu | Vaccine | Article | Community | Random sampling | 3,593 | Persons aged 60 years and older | May through August, 2007 | The United States | Yes |
| 7 | J.Y.C. Lau | International Journal of Infectious Diseases | Meeting abstract | Community | NA | 15 | Persons aged 65 years and older | NA | Hong Kong, China | No |
| 8 | Teba Al-Khalidi | Human Vaccines & Immunotherapeutics | Article | Community | Convenience sampling | 420 | Persons aged 50 years and older | February to April, 2019 | The United Arab Emirates | No |
| 9 | Xinyue Lu | Human Vaccines & Immunotherapeutics | Article | Community | Convenience sampling | 1,672 | Persons aged 50 years and older | Late 2020 | China | No |
| 10 | Louise A. Brown Nicholls | Vaccine | Article | Community | NA | 78 | Adults aged over 65 years | 8th February and 17th March, 2020 | The United Kingdom | No |
| 11 | Angela L. Funovits | Journal of the America Academy of Dermatology | Letter | Hospital | NA | 172 | NA | NA | The United States | No |
| 12 | Tae Un Yang | Human Vaccines & Immunotherapeutics | Article | Hospital | NA | 513 | Adults aged >18 years | August 23 and September 15, 2013 | South Korea | No |
| 13 | Jing Qiu | Chinese Journal of vaccines and immunization | Article | Hospital | Convenience sampling | 562 | Adults aged ≥50 years | May to June, 2020 | China | No |

NA: not applicable

**Table S2. Quality assessment of 13 included articles**

| **No.** | **Title and abstract** | **Introduction** | | **Methods** | | | | | | | | | **Results** | | | | | **Discussion** | | | | **Other information** | **Score** |
| --- | --- | --- | --- | --- | --- | --- | --- | --- | --- | --- | --- | --- | --- | --- | --- | --- | --- | --- | --- | --- | --- | --- | --- |
|  |  | Background/rationale | Objectives | Study design | Setting | Participants | Variables | Data sources/ measurement | Bias | Study size | Quantitative variables | Statistical methods | Participants | Descriptive data | Outcome data | Main results | Other analyses | Key results | Limitations | Interpretation | Generalisability | Funding |  |
| 1 | 1 | 1 | 1 | 1 | 1 | 1 | 1 | 1 | 0 | 0 | 1 | 1 | 0 | 1 | 1 | 1 | 0 | 1 | 1 | 1 | 1 | 0 | 17 |
| 2 | 1 | 1 | 1 | 1 | 1 | 0 | 0 | 0 | 0 | 0 | 0 | 1 | 0 | 1 | 1 | 1 | 0 | 1 | 0 | 0 | 0 | 0 | 10 |
| 3 | 1 | 1 | 1 | 1 | 1 | 1 | 1 | 1 | 0 | 1 | 1 | 1 | 0 | 1 | 1 | 1 | 0 | 1 | 0 | 1 | 0 | 1 | 17 |
| 4 | 1 | 1 | 1 | 1 | 1 | 1 | 1 | 1 | 0 | 1 | 1 | 1 | 0 | 1 | 1 | 1 | 1 | 1 | 1 | 1 | 1 | 1 | 20 |
| 5 | 1 | 1 | 1 | 1 | 1 | 1 | 1 | 1 | 0 | 1 | 1 | 1 | 1 | 1 | 1 | 1 | 1 | 1 | 1 | 1 | 1 | 0 | 20 |
| 6 | 1 | 1 | 1 | 1 | 1 | 1 | 1 | 1 | 0 | 1 | 1 | 1 | 0 | 1 | 1 | 1 | 0 | 1 | 1 | 1 | 0 | 0 | 17 |
| 7 | 1 | 1 | 1 | 0 | 1 | 1 | 0 | 0 | 0 | 0 | 0 | 0 | 0 | 1 | 1 | 1 | 0 | 1 | 0 | 1 | 0 | 0 | 10 |
| 8 | 1 | 1 | 1 | 1 | 1 | 1 | 1 | 1 | 0 | 1 | 1 | 1 | 1 | 1 | 1 | 1 | 0 | 1 | 1 | 1 | 1 | 1 | 20 |
| 9 | 1 | 1 | 1 | 1 | 1 | 1 | 1 | 1 | 0 | 0 | 1 | 1 | 0 | 1 | 1 | 1 | 0 | 1 | 1 | 1 | 1 | 1 | 18 |
| 10 | 1 | 1 | 1 | 1 | 1 | 1 | 1 | 1 | 0 | 0 | 1 | 1 | 0 | 1 | 1 | 1 | 0 | 1 | 0 | 1 | 0 | 1 | 16 |
| 11 | 1 | 1 | 1 | 0 | 0 | 0 | 1 | 1 | 0 | 0 | 0 | 0 | 0 | 1 | 1 | 1 | 0 | 1 | 0 | 0 | 0 | 1 | 10 |
| 12 | 1 | 1 | 1 | 1 | 1 | 1 | 1 | 1 | 1 | 0 | 1 | 1 | 1 | 1 | 1 | 1 | 0 | 1 | 1 | 1 | 1 | 1 | 20 |
| 13 | 1 | 1 | 1 | 1 | 1 | 1 | 1 | 1 | 0 | 0 | 1 | 1 | 0 | 1 | 1 | 1 | 0 | 1 | 1 | 1 | 0 | 1 | 17 |

**Table S3. Summarized influencing factors explored in 13 included articles**

| **Reference** | **Influencing factors** |
| --- | --- |
| 1 | Age §, Marital status (in a relationship/married vs single) †, Gender (male vs female) †, Education level §, Origin (private laboratory vs geriatric care department) †, Personal history of HZ §, Treatment of HZ §, People who know someone with a history of HZ §, People who think HZ causes pain (yes vs no) †, People who think HZ is always severe (yes vs no) †, Personal history of PHN §, Treatment of PHN §, Impairment in activities of daily living due to PHN §, People who know HZ vaccine §, People who think vaccination is a good prevention tool against HZ(yes vs no) †, People who agree to be vaccinated (all vaccines) (yes vs no) † |
| 2 | Patient’s general practitioner recommended (yes vs no) † |
| 3 | NA |
| 4 | Age (old vs young) † |
| 5 | Reasons to unwillingness: “They were unaware of its availability”, “Inadequate promotion from doctors and public education”, “Relatively high cost of the vaccine”, “Good self-perceived health” |
| 6 | Income (more vs less) †, Reasons to unwillingness: “The vaccination was not needed”, “Not thinking they were at risk”, “Not trusting doctors or medicine”, “Not knowing enough about the HZ vaccine”, “Concern about side effects” |
| 7 | Reasons to unwillingness: “No time at this visit”, “Can get vaccine cheaper elsewhere”, “Would like to postpone vaccination”, “Would like to address other medical problems first”, “General fear of vaccine”, “Would like to research vaccine”, “Concerned about interaction with other treatment”, “Would like to obtain PCP recommendations”, “Not covered by insurance”, “Believes vaccine is not important” |
| 8 | Age §, Gender §, Nationality §, Educational attainment §, Employment status §, Occupation §, Insured §, History of chronic diseases §, History of chickenpox (yes vs no sure) †, Reasons to unwillingness: “Not at risk since I am healthy”, “I do not believe in vaccines”, “prefers to take medication when sick”, “side effects of vaccine”, “financial” |
| 9 | Age (old vs young) ‡, Educational level §, Monthly income (more vs less) †, Knowing people in contact with those who have herpes zoster susceptible to contracting varicella-zoster virus (not susceptible vs susceptible) †, Knowing the elderly susceptible to contracting varicella-zoster virus (not susceptible vs susceptible) †, Knowing the recipients of the HZV vaccination in China (children vs adult ≥50 years) †, Knowing HZV vaccination schedule in China (not sure vs two doses) ‡, Vaccine hesitancy ‡ |
| 10 | Age §, Marital status §, Interpersonal support evaluation (more perceived support vs less perceived support) ‡, Vaccine hesitancy ‡ |
| 11 | NA |
| 12 | Age (old vs young) ‡, Gender §, Education (high vs low) †, Monthly household income §, personal history of HZ §, having witnessed someone with HZ §, self-reported rate of receiving the annual seasonal influenza vaccine §, Reasons to unwillingness: “Low perceived risk of developing HZ”, “Concerns about the adverse effects following immunization”, “Concerns about the vaccination cost”, “Misconceptions about the belief that after being infected once”, “no further vaccination is required”, “Fear of needles”, “Lack of physician’s recommendation of HZ vaccination”, “Uncertainty about the vaccine effectiveness” |
| 13 | Age (old vs young) ‡, Gender §, Education level (high vs low) †, Income (year) §, History of chronic diseases (yes vs no) ‡, Knowing HZ (yes vs no) †, Knowing HZ vaccine (yes vs no) †, knowing someone with a history of HZ (yes vs no) †, Perception of severity of HZ infection (yes vs no) †, Concerned about themselves infected with HZ (yes vs no) †, Believe vaccine can prevent HZ (yes vs no) † |

†: increase the willingness to be vaccinated, ‡: reduce the willingness to be vaccinated, §: no clear/no significant result, HZ: herpes zoster, PHN: postherpetic neuralgia; NA: not applicable.


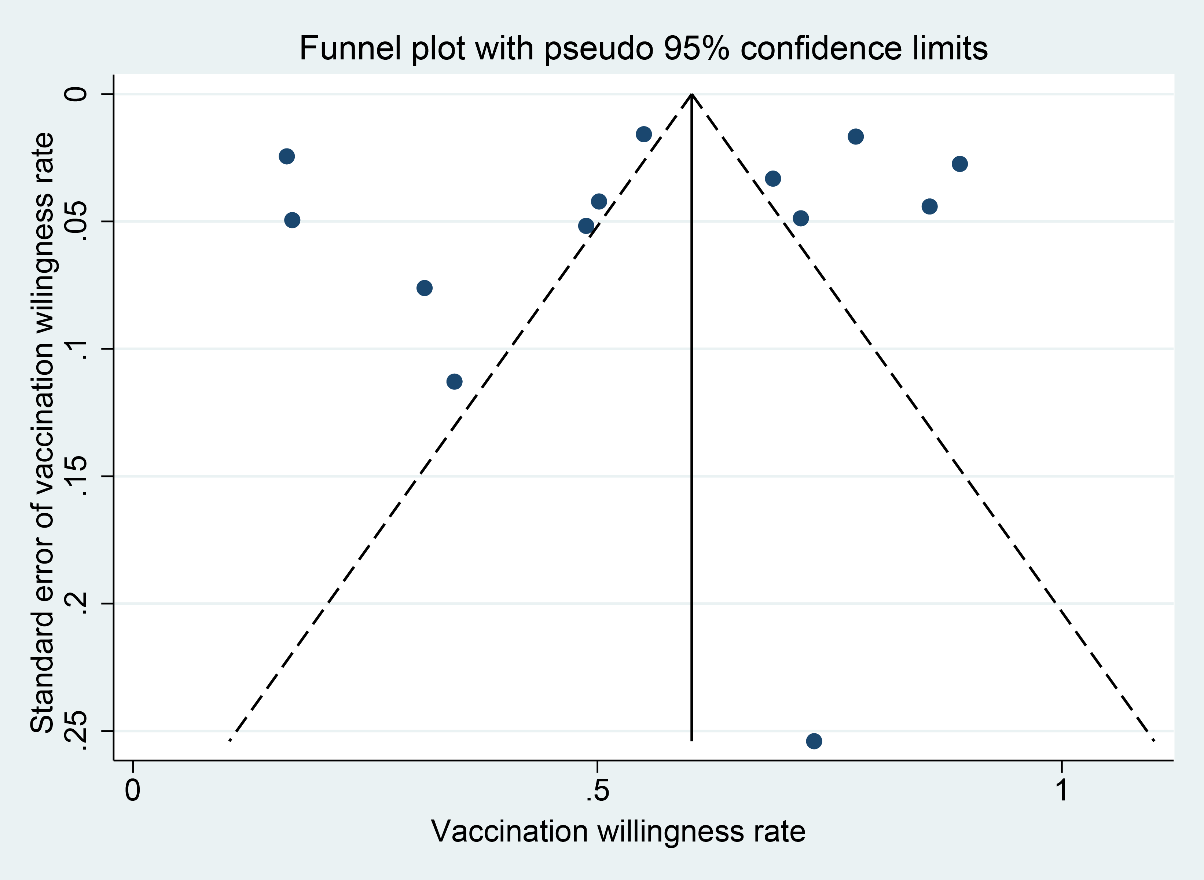


**Figure S1. Funnel plot**


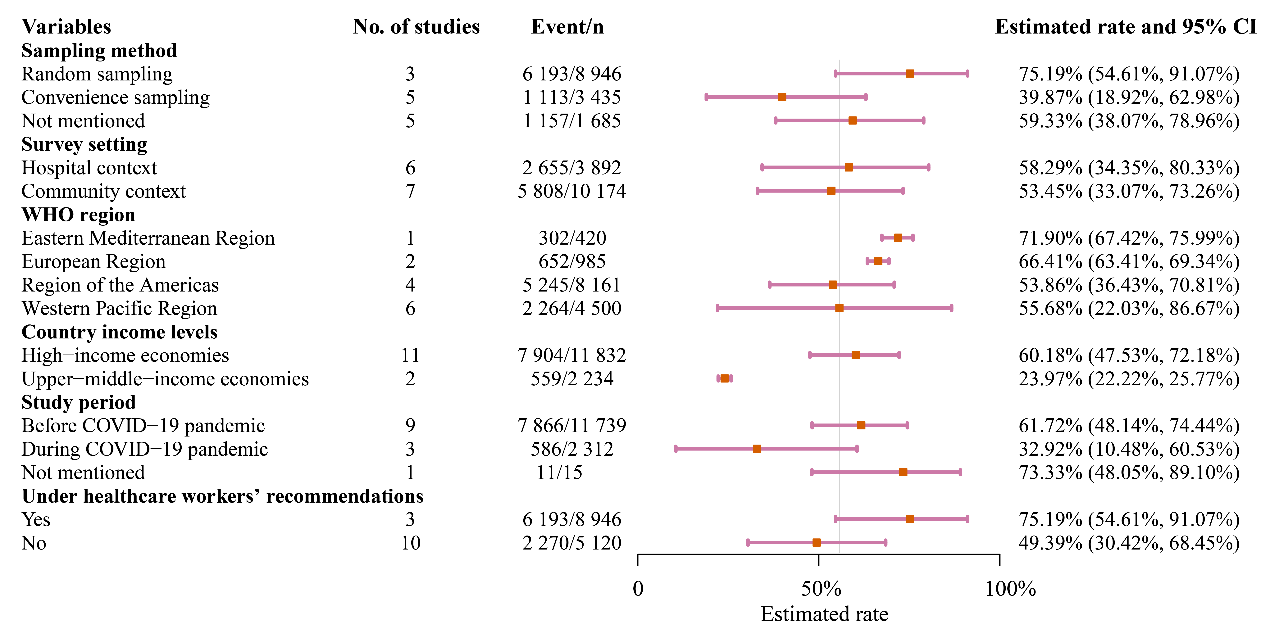


**Figure S2. Vaccination willingness rates among different subgroups ^a^**

^a^ The vertical bar represents the overall pooled rate (55.74%).


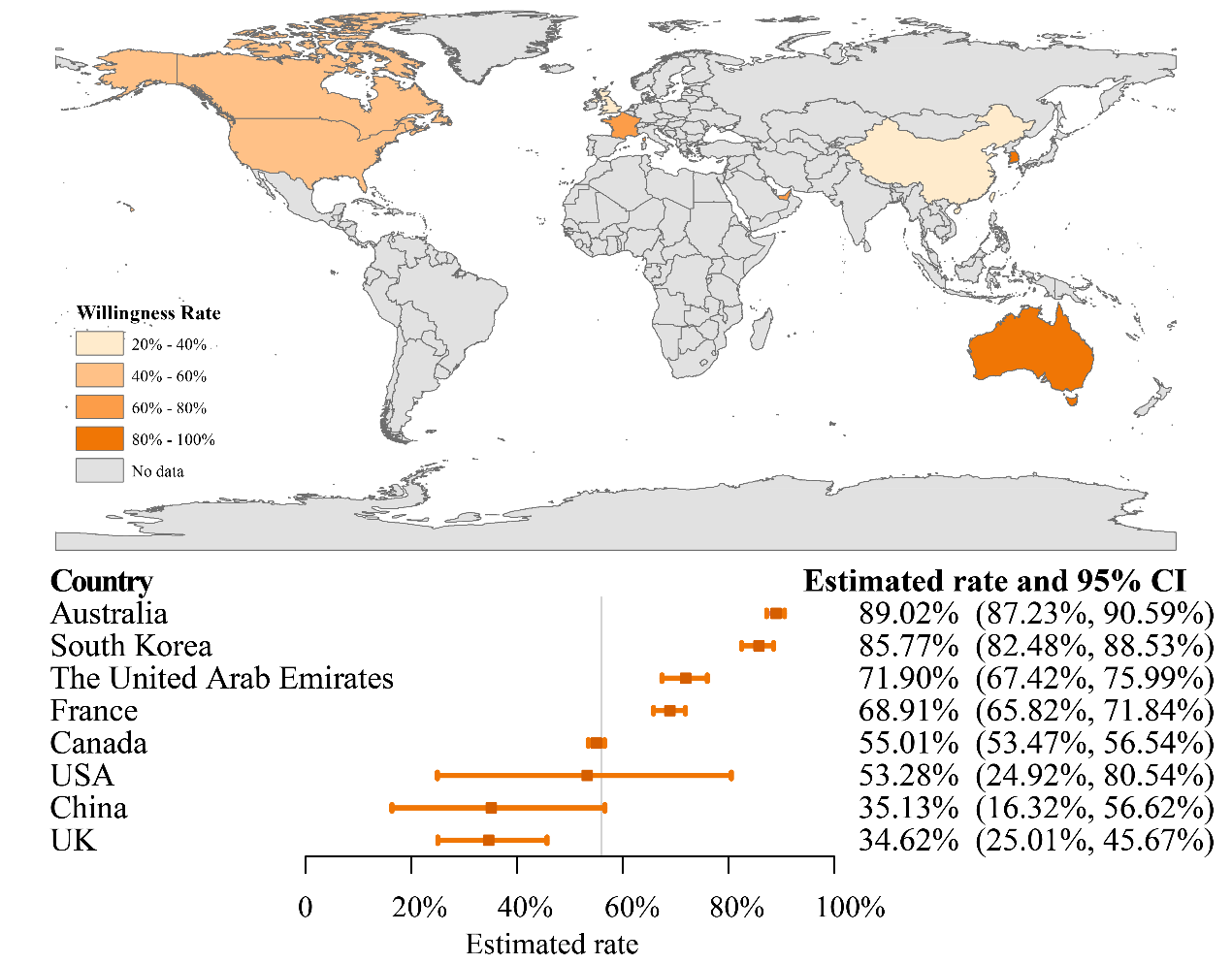


**Figure S3. Estimated HZ vaccination willingness rate worldwide ^a^**

^a^ The vertical bar represents the overall pooled rate (55.74%).

**Reference**

[1] Del Signore C, Hemmendinger A, Khanafer N, et al. Acceptability and perception of the herpes zoster vaccine in the 65 and over population: A French observational study. *Vaccine*. 2020;38(37):5891-5895. doi:10.1016/j.vaccine.2020.07.004

[2] Litt J.C.B., Kim S, Woodman R, Macintyre R, Cunningham T. Australian zoster study: GP and patient views about herpes zoster (shingles), its complications, and the likely acceptance of a zoster vaccine (Zostavax). *Int J Infect Dis*. 21. 436-437.doi: 10.1016/j.ijid.2014.03.1320

[3] MacDougall DM, Halperin BA, MacKinnon-Cameron D, et al. The challenge of vaccinating adults: attitudes and beliefs of the Canadian public and healthcare providers. *BMJ Open*. 2015;5(9):e009062. doi:10.1136/bmjopen-2015-009062

[4] Baalbaki NA, Fava JP, Ng M, et al. A Community-Based Survey to Assess Knowledge, Attitudes, Beliefs and Practices Regarding Herpes Zoster in an Urban Setting. *Infect Dis Ther*. 2019;8(4):687-694. doi:10.1007/s40121-019-00269-2

[5] Lam AC, Chan MY, Chou HY, et al. A cross-sectional study of the knowledge, attitude, and practice of patients aged 50 years or above towards herpes zoster in an out-patient setting. *Hong Kong Med J*. 2017;23(4):365-373. doi:10.12809/hkmj165043

[6] Lu PJ, Euler GL, Jumaan AO, Harpaz R. Herpes zoster vaccination among adults aged 60 years or older in the United States, 2007: uptake of the first new vaccine to target seniors. *Vaccine*. 2009;27(6):882-887. doi:10.1016/j.vaccine.2008.11.077

[7] Lau JYC., and Lee SS. Impedance of vaccination against influenza, pneumococcus and zoster among older adults in Hong Kong: A qualitative study. Int J Infect Dis. 101. 480-480. doi: 10.1016/j.ijid.2020.09.1256

[8] Al-Khalidi T, Genidy R, Almutawa M, et al. Knowledge, attitudes, and practices of the United Arab Emirates population towards Herpes Zoster vaccination: A cross-sectional study. *Hum Vaccin Immunother*. 2022;2073752. doi:10.1080/21645515.2022.2073752

[9] Lu X, Lu J, Zhang F, et al. Low willingness to vaccinate against herpes zoster in a Chinese metropolis. *Hum Vaccin Immunother*. 2021;17(11):4163-4170. doi:10.1080/21645515.2021.1960137

[10] Nicholls LAB, Gallant AJ, Cogan N, Rasmussen S, Young D, Williams L. Older adults’ vaccine hesitancy: Psychosocial factors associated with influenza, pneumococcal, and shingles vaccine uptake. *Vaccine*. 2021;39(26):3520-3527. doi:10.1016/j.vaccine.2021.04.062

[11] Funovits AL, Wagamon KL, Mostow EN, Brodell RT. Refusal of shingles vaccine: implications for public health. *J Am Acad Dermatol*. 2012;66(6):1011-1012. doi:10.1016/j.jaad.2011.11.931

[12] Yang TU, Cheong HJ, Song JY, Noh JY, Kim WJ. Survey on public awareness, attitudes, and barriers for herpes zoster vaccination in South Korea. *Hum Vaccin Immunother*. 2015;11(3):719-726. doi:10.1080/21645515.2015.1008885

[13] Qiu J, Sun XD, Hu, JY, Huang ZY, Guo X, Liang XF. Willingness to receive herpes zoster vaccine and factors influencing willingness among ⩾50-yearold adults of Shanghai in May-June 2020. *Chinese Journal of Vaccines and Immunization*. 2021;27(3) 307-310.
